# Plasma Natriuretic Peptides in Children and Adolescents with Obstructive Sleep Apnea and Their Changes Following Intervention

**DOI:** 10.3389/fped.2014.00022

**Published:** 2014-03-24

**Authors:** Albert Martin Li, Chun Ting Au, Jodie Y. Zhu, Kate Ching Ching Chan, Michael Ho Ming Chan, Dennis Lip Yen Lee, Yun Kwok Wing

**Affiliations:** ^1^Department of Paediatrics, The Chinese University of Hong Kong, Hong Kong, China; ^2^Department of Chemical Pathology, The Chinese University of Hong Kong, Hong Kong, China; ^3^Department of Otorhinolaryngology – Head and Neck Surgery, The Chinese University of Hong Kong, Hong Kong, China; ^4^Department of Psychiatry, Shatin Hospital, The Chinese University of Hong Kong, Hong Kong, China

**Keywords:** adenotonsillectomy, natriuretic peptides, obesity, obstructive sleep apnea

## Abstract

**Objective:** This study aimed to evaluate circulating natriuretic peptides (NP) concentration in obese and non-obese children and adolescents with and without obstructive sleep apnea (OSA), and their levels following OSA treatment.

**Methods:** Subjects with habitual snoring and symptoms suggestive of OSA were recruited. They underwent physical examination and overnight polysomnography (PSG). OSA was diagnosed if obstructive apnea–hypopnea index (OAHI) was ≥1/h. Fasting serum atrial natriuretic peptide (ANP) and brain natriuretic peptide (BNP) were taken after overnight PSG. The subjects were divided into obese, non-obese, with and without OSA groups for comparisons.

**Results:** One hundred fourteen children (77 were boys) with a median [interquartile range (IQR)] age of 10.8 (8.3–12.7) years (range: 2.4–11.8 years) were recruited. Sixty-eight subjects were found to have OSA. NP levels did not differ between subjects with and without OSA in both obese and non-obese groups. Stepwise multiple linear regressions revealed that body mass index (BMI) *z*-score was the only independent factor associated with NP concentrations. Fifteen children with moderate-to-severe OSA (OAHI >5/h) underwent treatment and there were no significant changes in both ANP and BNP levels after intervention.

**Conclusion:** Body mass index rather than OSA was the main determinant of NP levels in school-aged children and adolescents.

## Introduction

Obstructive sleep apnea (OSA) is characterized by repeated episodes of complete or partial upper airway occlusion during sleep. Childhood OSA if remains untreated can lead to behavioral changes and abnormalities in blood pressure control and ventricular function (1–4). This condition is increasingly recognized, and the prevalence in children is around 5% (5).

Both atrial natriuretic peptide (ANP) and brain natriuretic peptide (BNP) are neuro-hormones released by the cardiac ventricles and play an essential role in electrolytes regulation and water balance (6). Increased NP concentrations can decrease heart rate and blood pressure by regulating sympathetic activity and the renin–angiotensin–aldosterone pathway (7). These hormones may play an important role in OSA-related cardiovascular abnormalities. However, studies that examined ANP and BNP levels in adults with OSA have reported conflicting results (8–11). Maillard et al. documented a significant negative correlation between ANP and OSA severity (8). BNP levels were found to reduce markedly after the initiation of continuous positive airway pressure (CPAP) therapy (9). On the other hand, Svatikova et al. found no significant difference in both ANP and BNP levels in adult subjects with and without OSA (10). Similarly, Patwardhan et al. reported no age- and sex-adjusted significant difference in BNP between OSA adults and normal healthy subjects (11). Furthermore, Maeder et al. did not find significant association between OSA severity and a prohormone of BNP at baseline and after initiation of CPAP treatment (12, 13). Similar research in pediatrics is limited and the results are also contradictory. Kaditis et al. (14) reported no significant difference in evening or morning BNP levels between children with and without OSA while Sans-Capdevila et al. (15) documented higher plasma BNP levels in OSA children. A preliminary intervention study showed significant decrease in BNP following adenotonsillectomy in children with OSA (16). Further studies were therefore needed to better delineate the relationship between OSA and natriuretic peptide (NP) levels.

The aims of this study were to compare plasma concentrations of ANP and BNP between obese and non-obese children and adolescents with and without OSA, and to determine whether treatment for OSA would alter their levels. We hypothesized that body mass rather than OSA severity was the main determining factor associated with NP levels.

## Materials and Methods

### Subjects

Children with habitual snoring and symptoms suggestive of OSA were consecutively recruited from our Pediatric Respiratory, Sleep Disorder, and Obesity Clinic. Patients were excluded from the study if they were active smokers (self-reporting and confirmed by parents), had inter-current upper respiratory tract or systemic infection within 4 weeks of recruitment, suffered from neuromuscular disorder such as Duchenne muscular dystrophy, craniofacial anomalies, cardiovascular diseases, syndromic disorder, for example, Down syndrome, or if they had previously undergone upper airway surgery. Each subject and their parent completed a sleep-disordered breathing questionnaire on nighttime and daytime symptoms of OSA and past personal medical history (17). The study was approved by the Clinical Research Ethics Committee of the Chinese University of Hong Kong and informed written consent from the parents was obtained at the beginning of the assessment.

### Anthropometry and physical assessment

The weight and standing height of the subjects were measured with a calibrated weighing scale and stadiometer by standard anthropometric methods (18). Body mass index (BMI) was calculated as weight/height^2^ (kg/m^2^) and *z*-score derived using local reference (19). Children were defined as obese if their BMI *z*-score was ≥1.65, corresponding to the 95th percentile, relative to age and gender. Resting systolic blood pressure (SBP) and diastolic blood pressure (DBP) were measured by an oscillometric device (Accutorr Plus, Datascope Inc., Montvale, NJ, USA) with the subject sitting after 15 min rest. Three consecutive measurements were taken at 5-min intervals and average of the three readings was recorded.

### Polysomnography

An overnight polysomnography (PSG) was performed on each subject. Siesta ProFusion II PSG system (Compumedics Telemed PTY Ltd. Abbotsford, Australia) was used to record the following parameters: electroencephalogram from four leads (C_3_/A_2_, C_4_/A_1_), bilateral electrooculogram, electromyogram of mentalis activity, and bilateral anterior tibialis. Respiratory movements of the rib cage and abdomen were measured by pneumatic effort belt. Electrocardiogram and heart rate were continuously recorded from two anterior chest leads. Arterial oxyhemoglobin saturation (SaO_2_) was monitored by an oximeter (Ohmeda Biox 3900 Pulse Oximeter). Respiratory airflow pressure signals were measured at the anterior nares and connected to a pressure transducer. Snoring was assessed by a snoring microphone placed near the throat. Body position was recorded by a body position sensor. Obstructive apnea (OA) was defined as absence of airflow with persistent respiratory effort lasting longer than two baseline breaths, irrespective of SaO_2_ changes. Obstructive apnea index (OAI) was defined as the number of OA per hour of sleep. Hypopnea was defined as a reduction of 50% or more in the amplitude of the airflow signal. It was only quantified if longer than two baseline breaths and associated with oxygen desaturations of at least 3% and/or arousals. Obstructive apnea–hypopnea index (OAHI) was defined as the total number of obstructive apneic and hypopneic episodes per hour of sleep. Arousal was defined by standard criteria (20). We defined OSA as OAHI ≥1 episode per hour of sleep (21). Subjects who had an OAHI value <1 episode per hour of sleep were grouped as non-OSA.

### Laboratory measurements

All subjects had fasting blood samples taken in the morning following overnight PSG. Plasma proANP(1–98) was measured by enzyme-linked immunosorbent assay (Biomedica Medizinprodukte GmbH & Co KG, Wien, Divischgasse 4, Germany) (22) and BNP was measured by chemiluminescent immunoassay using the ADVIA Centaur Analyzer (Siemens Healthcare Diagnostics, Deerfield, IL, USA) (23). The detection limit and the inter-assay coefficients of variation for proANP and BNP were 50 pmol/L, 7.2% at 436 and 0.58 pmol/L, 1.9% at 8.5 pmol/L, respectively.

### Treatment and follow-up

Subjects with moderate-to-severe OSA, defined as OAHI >5/h, were offered adenotonsillectomy after assessment by an otorhinolaryngologist. Patients who refused surgery or in whom surgical intervention was not indicated as defined by pre-determined criteria (small tonsils; tonsils that do not extend beyond the anterior tonsillar pillar and small adenoids; adenoids, which occupy <25% of post-nasal space with minimal OSA symptoms or poorly controlled allergic rhinitis with supine nasal obstruction) were offered alternative treatment options including CPAP and/or nasal corticosteroids therapy (24). All subjects underwent repeat assessment 12 weeks after the operation or commencement of CPAP and or nasal corticosteroids. Subjects with mild OSA (OAHI 1–5/h) were followed-up in our sleep disorder clinic to monitor their disease progression but no further blood sampling was carried out.

### Statistical analysis

The subjects were divided into four groups (non-obese without OSA, non-obese with OSA, obese without OSA, and obese with OSA). Mean ± SD, median [interquartile range (IQR)], and number (percentage) were presented for parametric, non-parametric, and categorical data, respectively. Parametric and non-parametric data were compared using one-way analysis of variance (ANOVA) and the Kruskal–Wallis test, respectively. For parametric data, Tukey or Games–Howell method (depending on the agreement of the assumption of variance) was used for *post hoc* pairwise comparisons. For non-parametric data, Mann–Whitney *U* tests with adjusted *p* values (Bonferroni correction, significance at *p* < 0.05/6 pairwise comparisons = 0.008) were used for pairwise comparisons. The Chi-square test was used to assess the differences in proportion between the four groups with Bonferroni correction. Stepwise multiple regression analyses were used to assess the correlates of ANP and BNP. As BNP data were right-skewed, the data was log-transformed to obtain a normal distribution before entering into regression models. The following variables were put into the stepwise regression analyses for selection: age, gender, BMI *z*-score, OAI, OAHI, and arousal index. Two-way ANOVA was used to assess the interactive effect of obesity and OSA on NP. For the comparison of parameters before and after treatment of OSA, Wilcoxon signed-rank test was used to determine the differences. SPSS for Windows 14 (SPSS, Inc., Chicago, IL, USA) was used in the analysis.

## Results

One hundred fourteen subjects were invited and all agreed to participate in this study. There were 77 boys and the median (IQR) age of the subjects was 10.8 (8.3–12.7) years. The demographic characteristics, laboratory results, and PSG parameters were similar between boys and girls. All subjects had normal physical examination.

Sixty-eight subjects satisfied the diagnostic criteria for OSA and 42 of them were boys. The clinical characteristics of obese and non-obese subjects with and without OSA were compared in Table 1. Obese subjects had lower plasma ANP and BNP levels than non-obese subjects in both non-OSA and OSA groups, though this comparison did not reach statistical significance amongst children with OSA (Table 1). ANP and BNP levels were not different between OSA and non-OSA subjects in both obese and non-obese subgroups. Stepwise multiple regression analysis identified BMI *z*-score as the only factor significantly associated with ANP [β(SE) = −224.0(52.6), *p* < 0.001] and log-BNP [β(SE) = −0.24(0.09), *p * = 0.007]. The interactive effect of BMI *z*-score and OAHI on plasma ANP and BNP concentration was not significant (*p * = 0.588 and *p * = 0.914, respectively).

**Table 1:** **Demographic, laboratory, and sleep parameters of the subjects**.

	Non-obese		Obese		*p*
	Non-OSA (group 1)	OSA (group 2)	Non-OSA (group 3)	OSA (group 4)	
	(*n* = 26)	(*n* = 30)	(*n* = 20)	(*n* = 38)	
Age (years)	9.1 ± 3.1	9.9 ± 4.3	11.4 ± 3.2	11.2 ± 3.2	0.056
Male gender, *n* (%)	19 (73.1)	20 (66.7)	16 (80.0)	22 (57.9)	0.334
Weight (kg)^c^	29.3 ± 10.1	34.9 ± 17.8	63.7 ± 16.3	61.5 ± 21.4	<0.001
Height (m)^b^	1.32 ± 0.16	1.34 ± 0.24	1.51 ± 0.14	1.47 ± 0.15	<0.001
BMI *z*-score^c^	−0.004 ± 0.926	0.297 ± 1.051	2.209 ± 0.313	2.309 ± 0.472	<0.001
SBP (mmHg)^c^	101 ± 12	111 ± 17	121 ± 13	123 ± 16	<0.001
DBP (mmHg)	61 ± 9	65 ± 8	68 ± 6	70 ± 9	0.002
OAI/h^a,d^	0 (0–0)	1.7 (0.5–7.1)	0 (0–0.1)	1.0 (0.2–4.6)	<0.001
OAHI/h^a,d^	0.2 (0–0.5)	9.4 (4.2–19.8)	0.2 (0.1–0.5)	4.5 (2.0–14.2)	<0.001
Arousal index/h^a^	5.7 (3.9–9.9)	8.9 (6.6–14.5)	5.3 (4.0–7.8)	6.0 (4.4–13.6)	0.007
ANP (fmol/ml)^b^	2292 ± 948	2026 ± 656	1467 ± 655	1660 ± 658	<0.001
BNP (pmol/l)	3.1 (1.6–7.6)	3.7 (1.6–9.6)	1.4 (0.3–5.3)	2.8 (0.7–5.4)	0.067

*BMI, body mass index; SBP, systolic blood pressure; DBP, diastolic blood pressure; OAI, obstructive apnea index; OAHI, obstructive apnea–hypopnea index; ANP, atrial natriuretic peptide; BNP, brain natriuretic peptide*.

*Data were presented as mean ± SD, median (IQR), and number (percentage) for parametric, non-parametric, and categorical data, respectively*.

*p values were obtained from ANOVA, Kruskal–Wallis test, and Chi-square test table for parametric, non-parametric, and categorical data, respectively*.

*Post hoc analyses, Tukey HSD, or Games–Howell method for the parametric variables and Mann–Whitney *U* test with adjusted *p* values (significant at *p * < 0.008) for non-parametric variables*.

*^a^Significant difference between group 1 and 2*.

*^b^Significant difference between group 1 and 3*.

*^c^Significant difference between group 2 and 4*.

*^d^Significant difference between group 3 and 4*.

Of the 68 OSA subjects, 39 had an OAHI >5/h and of whom 15 agreed for treatment at our institution and returned for re-assessment. Twelve had adenotonsillectomy and one obese subject was started on CPAP. Two subjects who had small tonsils but reported to have allergic rhinitis symptoms were given 3 months of nasal spray corticosteroids. Twenty-four subjects did not undergo repeat assessment. There were, however, no significant differences in demographic characteristics, laboratory results, and PSG parameters between those who had repeat assessment and those who did not. For the 15 subjects who returned for repeat assessment, 2 had missing BNP level data. There were no significant changes in ANP and BNP concentrations following intervention for OSA (Table 2).

**Table 2:** **Laboratory and PSG parameters of the 15 subjects before and after treatment**.

	Pre	Post	*p*
Age (years)	10.8 (6.6–13.3)	11.1 (7.4–14.8)	<0.001
Weight (kg)	49.5 (18.0–64.1)	51.4 (20.7–72.6)	0.073
Height (m)	1.45 (1.15–1.60)	1.53 (1.19–1.61)	0.010
Body mass index (kg/m^2^)	21.1 (15.2–25.6)	22.8 (15.9–26.3)	0.112
Body mass index *z*-score	1.20 (−0.18–1.91)	1.40 (0.17–1.83)	0.394
Systolic blood pressure (mmHg)	111 (102–129)	111 (103–126)	0.432
Diastolic blood pressure (mmHg)	67 (59–70)	63 (60–71)	0.636
Obstructive apnea index/h	7.8 (1.2–13.7)	0 (0–0)	<0.001
Obstructive apnea–hypopnea index/h	19.5 (9.6–33.3)	0.8 (0.5–1.5)	<0.001
Arousal index/h	9.1 (6.3–22.2)	4.8 (2.6–7.8)	<0.001
ANP (fmol/ml)	1861 (1121–2289)	1528 (968–2055)	0.112
BNP (pmol/l) (*n* = 13)	4.1 (1.3–7.0)	2.5 (0.5–5.4)	0.173

*Data were presented as median (IQR). *p* values were obtained from Wilcoxon signed-rank tests*.

*ANP, atrial natriuretic peptide; BNP, brain natriuretic peptide*.

## Discussion

In this study, we failed to demonstrate any relationship between NP levels and OSA severity at both baseline and following intervention for OSA. We found significantly lower plasma ANP and BNP levels in obese than non-obese subjects for the non-OSA group. Furthermore, a trend of lower plasma NP levels in obese subjects was also observed for children with OSA. BMI *z*-score rather than OSA severity was the only independent predictor for ANP and BNP levels.

Atrial natriuretic peptide and BNP are NPs produced by myocytes of the ventricles and they are released in response to increased cardiac pressure and volume load (25). Children with heart failure or pulmonary hypertension have increased ANP and BNP levels (26). NP exert potent lipolytic effects in isolated human fat cells and adipocytes (27). Birkenfeld et al. documented that ANP could activate hormone-sensitive lipase in human fat cells and leads to lipolysis (28). Therefore, increased NP could inhibit lipid accumulation in adipose tissue and skeletal muscle and decrease cardiovascular risk (29).

The current literature examining NP in subjects with OSA has conflicting results. In our study, both ANP and BNP were documented to be closely associated with BMI only. We failed to show different NP levels in OSA and non-OSA subjects. Furthermore significant association between their plasma levels with variables of OSA such as OAHI, oxygen saturation nadir, and arousal index was not demonstrated in regression analysis. Our result was consistent with findings reported by Kaditis et al. (14) who showed no significant difference in morning and evening BNP levels between children with OSA and normal controls, though they did find a significantly greater overnight increase in BNP levels amongst subjects with moderate-to-severe OSA. In contrast, Sans-Capdevila et al. (15) documented higher morning BNP levels in OSA children compared with normal controls. Different age range, inclusion of obese subjects, and milder OSA disease amongst our subjects in our study could explain the discrepancy. The use of snoring non-OSA subjects for comparison may be another reason to explain why we failed to show significant differences in NP levels between our study groups. In the study by Sans-Capdevila et al. the authors also failed to show significant difference in BNP levels between OSA and subjects with primary snoring (15). There is on-going argument related to whether primary snoring is indeed a benign entity. In our previous publications, we found higher blood pressure and abnormal endothelial function in children with primary snoring compared to healthy controls (30, 31). Therefore, using primary snorers as a comparison group may dilute the overall difference in ANP and BNP levels.

Lower ANP and BNP levels were found in obese subjects. Our results were consistent with a previous study on NP and obesity in children where lower BNP levels were documented in obese children (32). Similar results have also been reported in several adult studies (33–35). NP possess lipolytic properties (27) and hence it might play a role in weight loss or weight maintenance. Furthermore, adipose tissue is a source of NP clearance receptors, therefore, increase in adipose tissue increases the clearance of NP and low NP levels would be expected (36).

Following intervention for OSA, no significant change in both ANP and BNP levels were found. However a previous study conducted in children with OSA demonstrated a significant reduction in BNP levels 4–6 months after adenotonsillectomy. The discrepancy may be attributed to our shorter follow-up period (16). Another study showed that BNP levels decreased significantly after adenotonsillectomy in young children with OSA (37). The age range of subjects in the study was much lower compared to our cohort. Their mean age was 19.7 months. Furthermore, the subjects had more severe OSA disease, their mean AHI was 16.9/h. In an adult study that involved patients with OSA with and without cardiovascular disease, the authors did not find significant changes in ANP or BNP levels following CPAP (13).

There were several limitations in our study. First, we managed to obtain post-intervention data in only 15 out of 39 OSA subjects. There were however, no significant differences in demographic, laboratory, and sleep apnea characteristics between those who underwent and those who did not have repeat assessment. Second, we could have selected a biased sample as we recruited our subjects from attendants to our Pediatric Respiratory, Sleep Disorder, and Obesity Clinic. Boys were more likely to be obese and this could explain the male preponderance seen in this study. Lastly, we did not have a control group of non-snoring children for comparison. However, it is practically difficult to recruit healthy non-snoring children for overnight sleep study and blood sampling.

In summary, our results demonstrated that weight rather than OSA was the important determining factor for ANP and BNP levels in both obese and non-obese school-age children.

## Conflict of Interest Statement

The authors declare that the research was conducted in the absence of any commercial or financial relationships that could be construed as a potential conflict of interest.

## References

[1] Lumeng JC Chervin RD Epidemiology of pediatric obstructive sleep apnea. Proc Am Thorac Soc (2008) 5:242–52 10.1513/pats.200708-135MG 18250218 PMC2645255

[2] Gozal D Sleep-disordered breathing and school performance in children. Pediatrics (1998) 102:616–20 10.1542/peds.102.3.616 9738185

[3] Li AM Au CT Sung RY Ho C Ng PC Fok TF Ambulatory blood pressure in children with obstructive sleep apnoea: a community based study. Thorax (2008) 63:803–9 10.1136/thx.2007.091132 18388205

[4] Chan JY Li AM Au CT Lo AF Ng SK Abdullah VJ Cardiac remodelling and dysfunction in children with obstructive sleep apnoea: a community based study. Thorax (2009) 64:233–9 10.1136/thx.2007.094904 19008295

[5] Li AM So HK Au CT Ho C Lau J Ng SK Epidemiology of obstructive sleep apnoea syndrome in Chinese children: a two-phase community study. Thorax (2010) 65:991–7 10.1136/thx.2010.134858 20965935

[6] Kuwahara K Nakao K Regulation and significance of atrial and brain natriuretic peptides as cardiac hormones. Endocr J (2010) 57:555–65 10.1507/endocrj.K10E-150 20571250

[7] Rubattu S Sciarretta S Valenti V Stanzione R Volpe M Natriuretic peptides: an update on bioactivity, potential therapeutic use, and implication in cardiovascular diseases. Am J Hypertens (2008) 21:733–41 10.1038/ajh.2008.174 18464748

[8] Maillard D Fineyre F Dreyfuss D Djedaini K Blanchet F Paycha F Pressure-heart rate responses to alpha-adrenergic stimulation and hormonal regulation in normotensive patients with obstructive sleep apnea. Am J Hypertens (1997) 10:24–31 10.1016/S0895-7061(96)00252-X 9008245

[9] Kita H Ohi M Chin K Noguchi T Otsuka N Tsuboi T The nocturnal secretion of cardiac natriuretic peptides during obstructive sleep apnoea and its response to therapy with nasal continuous positive airway pressure. J Sleep Res (1998) 7:199–207 10.1046/j.1365-2869.1998.00109.x 9785275

[10] Svatikova A Shamsuzzaman AS Wolk R Phillips BG Olson LJ Somers VK Plasma brain natriuretic peptide in obstructive sleep apnea. Am J Cardiol (2004) 94:529–32 10.1016/j.amjcard.2004.05.010 15325948

[11] Patwardhan AA Larson MG Levy D Benjamin EJ Leip EP Keyes MJ Obstructive sleep apnea and plasma natriuretic peptide levels in a community-based sample. Sleep (2006) 29:1301–6 17068983 10.1093/sleep/29.10.1301

[12] Maeder MT Ammann P Rickli H Schoch OD Korte W Hürny C N-terminal pro-B-type natriuretic peptide and functional capacity in patients with obstructive sleep apnea. Sleep Breath (2008) 12:7–16 10.1007/s11325-007-0143-9 17906885

[13] Maeder MT Ammann P Münzer T Schoch OD Korte W Hürny C Continuous positive airway pressure improves exercise capacity and heart rate recovery in obstructive sleep apnea. Int J Cardiol (2009) 132:75–83 10.1016/j.ijcard.2007.10.040 18191481

[14] Kaditis AG Alexopoulos EI Hatzi F Kostadima E Kiaffas M Zakynthinos E Overnight change in brain natriuretic peptide levels in children with sleep-disordered breathing. Chest (2006) 130:1377–84 10.1378/chest.130.5.1377 17099013

[15] Sans-Capdevila O Crabtree VM Kheirandish-Gozal L Gozal D Increased morning brain natriuretic peptide levels in children with nocturnal enuresis and sleep-disordered breathing: a community-based study. Pediatrics (2008) 121:e1208–14 10.1542/peds.2007-2049 18450864

[16] Kaditis AG Chaidas K Alexopoulos EI Varlami V Malakasioti G Gourgoulianis K Effects of adenotonsillectomy on R-R interval and brain natriuretic peptide levels in children with sleep apnea: a preliminary report. Sleep Med (2011) 12:646–51 10.1016/j.sleep.2011.01.014 21697008

[17] Li AM Cheung A Chan D Wong E Ho C Lau J Validation of a questionnaire instrument for prediction of obstructive sleep apnea in Hong Kong Chinese children. Pediatr Pulmonol (2006) 41:1153–60 10.1002/ppul.20505 17054110

[18] Tanner JM Physical growth and development. In: Forfar JO Arneil GC, editors. Textbook of Paediatrics. Edinburgh: Churchill Livingstone (1984). p. 304–5

[19] Leung SS Cole TJ Tse LY Lau JT Body mass index reference curves for Chinese children. Ann Hum Biol (1998) 25:169–74 10.1080/03014469800005542 9533516

[20] American Sleep Disorders Association EEG arousals: scoring rules and examples. A preliminary report from the Sleep Disorders Atlas Task Force of the American Sleep Disorders Association. Sleep (1992) 15:174–84 11032543

[21] Marcus CL Omlin KJ Basinki DJ Bailey SL Rachal AB Von Pechmann WS Normal polysomnographic values for children and adolescents. Am Rev Respir Dis (1992) 146:1235–9 10.1164/ajrccm/146.5_Pt_1.1235 1443877

[22] Silver MA Maisel A Yancy CW McCullough PA Burnett JC Jr Francis GS BNP Consensus Panel 2004: a clinical approach for the diagnostic, prognostic, screening, treatment monitoring, and therapeutic roles of natriuretic peptides in cardiovascular diseases. Congest Heart Fail (2004) 10:1–30 10.1111/j.1527-5299.2004.03271.x 15604859

[23] Valenti G Fraszl W Addabbo F Tamma G Procino G Satta E Water immersion is associated with an increase in aquaporin-2 excretion in healthy volunteers. Biochim Biophys Acta (2006) 1758:1111–6 10.1016/j.bbamem.2006.03.029 16764820

[24] Brouillette RT Manoukian JJ Ducharme FM Oudjhane K Earle LG Ladan S Efficacy of fluticasone nasal spray for pediatric obstructive sleep apnea. J Pediatr (2001) 138:838–44 10.1067/mpd.2001.114474 11391326

[25] Nakagawa O Ogawa Y Itoh H Suga S Komatsu Y Kishimoto I Rapid transcriptional activation and early mRNA turnover of brain natriuretic peptide in cardiocyte hypertrophy. Evidence for brain natriuretic peptide as an “emergency” cardiac hormone against ventricular overload. J Clin Invest (1995) 96:1280–7 10.1172/JCI118162 7657802 PMC185749

[26] Nir A Nasser N Clinical value of NT-ProBNP and BNP in pediatric cardiology. J Card Fail (2005) 11:S76–80 10.1016/j.cardfail.2005.04.009 15948106

[27] Galitzky J Sengenès C Thalamas C Marques MA Senard JM Lafontan M The lipid-mobilizing effect of atrial natriuretic peptide is unrelated to sympathetic nervous system activation or obesity in young men. J Lipid Res (2001) 42:536–44 11290825

[28] Birkenfeld AL Boschmann M Moro C Adams F Heusser K Franke G Lipid mobilization with physiological atrial natriuretic peptide concentrations in humans. J Clin Endocrinol Metab (2005) 90:3622–8 10.1210/jc.2004-1953 15741263

[29] Bergman RN Kim SP Hsu IR Catalano KJ Chiu JD Kabir M Abdominal obesity: role in the pathophysiology of metabolic disease and cardiovascular risk. Am J Med (2007) 120:S3–8 10.1016/j.amjmed.2006.11.012 17296343

[30] Li AM Au CT Ho C Fok TF Wing YK Blood pressure is elevated in children with primary snoring. J Pediatr (2009) 155:362–8 10.1016/j.jpeds.2009.03.041 19540515

[31] Li AM Au CT Chook P Lam HS Wing YK Reduced flow-mediated vasodilation of brachial artery in children with primary snoring. Int J Cardiol (2013) 167(5):2092–6 10.1016/j.ijcard.2012.05.108 22703940

[32] Pervanidou P Akalesto A Sakka S Kanaka-Gantenbein C Papassotiriou I Chrousos GP Gender dimorphic associations between N-terminal pro-brain natriuretic peptide, body mass index and blood pressure in children and adolescents. Horm Res Paediatr (2010) 73:341–8 10.1159/000308166 20389104

[33] Das SR Drazner MH Dries DL Vega GL Stanek HG Abdullah SM Impact of body mass and body composition on circulating levels of natriuretic peptides: results from the Dallas Heart Study. Circulation (2005) 112:2163–8 10.1161/CIRCULATIONAHA.105.555573 16203929

[34] Mehra MR Uber PA Park MH Scott RL Ventura HO Harris BC Obesity and suppressed B-type natriuretic peptide levels in heart failure. J Am Coll Cardiol (2004) 43:1590–5 10.1016/j.jacc.2003.10.066 15120816

[35] Wang TJ Larson MG Levy D Benjamin EJ Leip EP Wilson PW Impact of obesity on plasma natriuretic peptide levels. Circulation (2004) 109:594–600 10.1161/01.CIR.0000112582.16683.EA 14769680

[36] Sarzani R Paci VM Zingaretti CM Pierleoni C Cinti S Cola G Fasting inhibits natriuretic peptides clearance receptor expression in rat adipose tissue. J Hypertens (1995) 13:1241–6 10.1097/00004872-199511000-00004 8984120

[37] Goldbart AD Levitas A Greenberg-Dotan S Ben Shimol S Broides A Puterman M B-type natriuretic peptide and cardiovascular function in young children with obstructive sleep apnea. Chest (2010) 138:528–35 10.1378/chest.10-0150 20558551

